# Effect of *Nanog* overexpression on the metastatic potential of a mouse melanoma cell line B16-BL6

**DOI:** 10.1007/s11010-021-04110-8

**Published:** 2021-03-04

**Authors:** Mikako Saito, Ryota Kishi, Tomoko Sasai, Tomohiro Hatakenaka, Nahoko Matsuki, Seiya Minagawa

**Affiliations:** Department of Biotechnology and Life Science, Tokyo University of Agriculture and Technology, 2-24-16, Naka-cho, Koganei, Tokyo 184-8588 Japan

**Keywords:** *Nanog*, Undifferentiated state, Mouse melanoma, Metastasis, MMP9

## Abstract

*Nanog*, a marker and regulator of the undifferentiated state in embryonic stem cells were anticipated to be an effective enhancer of cancer metastasis. We have developed a *Nanog* overexpressing mouse melanoma cell line B16-BL6 (BL6). BL6 was well recognized as a cell line with a high metastatic potential. In vitro tests revealed the enhancement of cell proliferation, wound healing activity, and matrix metalloproteinase 9 (MMP9) activity. Nanog-induced up- or down-regulated genes were comprehensively analyzed by transcriptome sequencing using Nanog^+^BL6 and wild-type BL6. Principally, up-regulated genes were involved in vesicle-aided glucose transport and oxidative phosphorylation, while down-regulated genes were associated with immunosuppression and apoptosis. A marked finding was that *TGF-β1* was down-regulated, because *TGF-β1* has been well discussed about its suppressive/progressive dual role in cancer. In vivo test showed that the number and volume of metastatic colonies of BL6 to lung were as high as 115 colonies/lung and 5.6 mm^3^/lung. Under this condition, *Nanog* overexpression caused a progressive effect (150 colonies/lung, *p* = 0.25; 9.2 mm^3^/lung, *p* = 0.13) rather than a suppressive effect on the metastasis. In this study, the effectiveness of *Nanog* overexpression in enhancing the metastatic potential of melanoma cell lines has been demonstrated for the first time.

**Supplementary Information:**

The online version contains supplementary material available at 10.1007/s11010-021-04110-8.

## Introduction

Cancer cells initially appearing in normal tissues often change their properties as they grow. Not only do the size and density of a cell mass change, but drug resistance may also increase. Even though most of a cancer cell mass may appear to be removed by surgery or drug therapy, occasions may occur where a small number of residual cancer cells with high drug resistance remain. These cells eventually grow, migrate, and travel throughout the body to become metastatic colonies.

Despite a great number of studies, however, the activation mechanisms of such cancer cells are still unclear. Finding a novel enhancer of metastasis may lead to a clue to solve this problem. Here, we have selected *Nanog* as a candidate of such an enhancer, because of frequent suggestions that metastatic potential should increase as the cancer cell state becomes more undifferentiated. Such relevant factors include *Nanog*, *Oct3/4*, *Sox2*. Of these, we focused on *Nanog* since preliminary studies suggested that *Nanog* was a more effective enhancer of metastasis than other factors (unpublished data).

The overexpression of *Nanog* in A375 human melanoma cells induced an increase in invasion three times higher than that of the control [1], as demonstrated by an in vitro test. Delivery of si*Nanog* into tumor-bearing mice suppressed the growth of tumor cells [TC-1 (mouse lung), HEK293 (human embryonic kidney), and CaSki (human cervical)] [2], suggesting the in vivo tumor-promoting effect of *Nanog*. The metastatic potential of mammary tumors to the lungs in *Nanog* and *Wnt-1*-overexpressing mice was 5–6 times higher than that in *Wnt-1*-overexpressing mice [3], which highlighted the growth promotion, but not tumorigenesis, by *Nanog*. In ES-2 ovarian cancer cell lines, *Nanog* was controlled by *Hexokinase2* (*HK2*) [4]. Short hairpin RNA (sh*HK2*) in ES-2 cells injected subcutaneously into mice generated smaller tumors (42%) than control. The decrease in *HK2* caused a decrease in *Nanog* expression and, consequently, the tumor size decreased.

Conversely, however, there was an occasion that *Nanog* overexpression down-regulated metastasis. In *Nanog* overexpressing human bladder cancer cells, invasion was reduced from 0.56 to 0.11 μm/min [5]. In this case, *Nanog* down-regulated *thymosin β4* and *rho family GTPase-3*, suggesting the inhibition of actin-binding activity. A possible role of *Nanog* in suppressing metastasis was suggested by in vitro test.

Therefore, it is still unclear whether *Nanog* is a progressor or a suppressor of tumor growth and metastasis. Nevertheless, we presume that *Nanog* should be a strong progressor. This study was aimed at its assertion by demonstrating further enhancement of the metastatic potential of a cancer cell line with originally high metastatic potential. From this viewpoint, we have selected BL6 that is well recognized as a cell line with high metastatic potential.

## Materials and methods

### Cell culture

In wild type of mouse melanoma cell line, BL6 cells were cultured in R10 medium (RPMI-1640 [Gibco, Carlsbad, CA, USA] containing 10% fetal beef serum and 1% penicillin–streptomycin [Gibco]) maintained at 37 °C under 5% CO_2_. Cancer cells are thought to be sensitive to oxygen condition; therefore, hypoxic culture was also conducted under 0.7–1.0% O_2_ for comparative analysis. Cells were cultured under normoxic condition at 37 °C under 5% CO_2_ for 24 h and then successively under hypoxic condition at 37 °C under 5% CO_2_ for 48 h.

### Animals

C57BL/6 male mice were bred in a specific pathogen-free room under conditions of 12 h illumination and 12 h darkness each day. Every mouse was fed a solid diet (MF, Oriental Yeast Co., Ltd., Tokyo, Japan) for 8–9 weeks from birth. Animal experimental procedures were conducted according to the guidelines of the “Guide for the Care and Use of the Laboratory Animals” of Tokyo University of Agriculture and Technology and were approved by the Institutional Animal Care and Use Committee of Tokyo University of Agriculture and Technology (IACUC number 29-57, 30-128, 25-69).

### Preparation of a *Nanog* overexpressing melanoma cell line

An overexpression vector for *Nanog* was constructed by inserting the *Nanog* gene into a pCAG-IRES-PuroR-EGFP vector. The vector product (4 μg/250 μL RPMI) and a Lipofectamine 2000 solution (5 μL/250 μL RPMI) were gently mixed and then incubated at 25 °C for 20 min. This mixture was added to 90% confluent melanoma cells and incubated for 4 h. Subsequently, the cells were cultured in fresh R10 medium at 37 °C for 24 h. After replacing the medium with R10 containing 1.5 μg/mL puromycin, cells were cultured for 2 weeks to select *Nanog* overexpressing BL6 (*Nanog*^+^BL6) cells. The introduction of *Nanog* was confirmed by reverse transcriptase (RT)-PCR and western analysis as described below.

### Measurement of cell proliferation

BL6 and *Nanog*^+^BL6 cells were cultured in R10 medium in dishes (6 cm^ϕ^), respectively. The initial cell density was 1 × 10^5^ cells/dish. Replacing the medium with fresh R10 at 48 h, the culture was continued for another 48 h. Cells were collected at 48 h and 98 h by trypsin/EDTA treatment, and cells number was determined.

### Wound healing assay

BL6 cells and *Nanog*^+^BL6 cells were cultured, respectively, in R10 medium in dishes (6 cm^ϕ^) at 37 °C under 5% CO_2_. The initial cell density was 2 × 10^5^ cells/dish. After replacing the medium at 24 h with fresh R10, culturing was continued for another 24 h to obtain 100% confluent cells. A uniform scratch was made down the center of each dish using a sterile micropipette tip, and scraped cells were removed by washing with phosphate buffered saline (PBS). After adding R10 medium, the culture was continued for 24 h. During this culture, the wound area between the two boundaries of the cellular area became narrower. The wound area was measured at 0, 4, 6, 12, and 24 h by means of ImageJ software.

### Quantitative RT-PCR

Total RNA was prepared using ISOGEN II (Nippongene, Tokyo, Japan) according to the manufacturer’s instructions. According to a protocol described previously [6], the expression levels of *Nanog*, *MMP2*, *MMP9*, *TGF-β1* mRNA were analyzed by quantitative (q)RT-PCR using a StepOnePlus™ Real-Time PCR System (Applied Biosystems) under the following conditions: 95 °C for 10 min, 45 cycles of a reaction set (95 °C denaturation for 15 s, 60 °C annealing for 1.0 min), and a reaction set for melt curve analysis (95 °C for 15 s and 60 °C for 1 min). Primer sets and expected product sizes of respective target RNAs are listed in a supplemental table (S1 Table). The amount of target mRNA was normalized to the amount of *Gapdh* mRNA.

The expression levels of *Nanog*, *HIF-1α* (hypoxia inducible factor), and *TGF-β1* were investigated also under hypoxic culture condition. The amount of target mRNA was normalized to the amount of *RPL13α* mRNA that was used as the internal standard in the study under hypoxic condition [7]. Primer set and expected product sizes of target RNAs of *HIF-1α* and *RPL13α* are included in S1 Table.

### Transcriptome sequencing analysis

Differential transcriptional activities of BL6 and *Nanog*^+^BL6 cells were analyzed by transcriptome sequencing entrusted to Genewiz (Kawaguchi, Japan).

The experimental workflow was RNA extraction and quality control (Agilent bioanalyzer 2100), library construction (NEBNext® Ultra RNA Library Prep Kit for Illumina), purification (Beckman Agencourt AMPure XP beads), library QC and quantification (Agilent bioanalyzer 2100 & Qubit), sequencing cluster generation (TruSeq PE Cluster Kit V4), and high throughput sequencing (TruSeqSBS Kit V4-HS, Illumina HiSeq platform).

Bioinformatics workflow was sequencing data quality assessment and filtering, data alignment to reference genome, alternative splicing analysis, novel transcript prediction, genetic mutation (SNV, InDel) analysis, gene expression analysis, RNA-seq overall quality assessment, gene differential expression analysis, differential gene ontology (GO) enrichment analysis, and KEGG enrichment analysis [8, 9]. The involvement of up-regulated and down-regulated genes in the most important biochemical metabolic pathways and signal transduction pathways was analyzed based on the KEGG.

### Western analysis

Protein sample solutions were prepared from melanoma cells. The protein concentration was determined using a Pierce® BCA™ Protein Assay kit (Thermo Fisher Scientific). A sample solution containing 30 μg protein was separated by SDS-PAGE.

Blotting onto a PVDF membrane was conducted at 100 V for 3 h at 4 °C. The PVDF membrane was then immersed in 5% skim milk for 30 min. Next, the PVDF membrane was incubated in a 5% skim milk containing anti-mouse *Nanog* antibody (C-4, 1:500, Santa Cruz Biotechnology, Inc., Dallas, TX, USA) and anti-mouse Gapdh (6C5, 1:1000, Santa Cruz Biotechnology) for 2 h at 25 °C.

After the reaction with primary antibodies, the PVDF membrane was washed with TBS-T (0.25 M Tris-HCl buffer (pH 7.4), 1.5 M NaCl, 0.01% Tween 20) for 5 min and then incubated in TBS-T containing anti-mouse immunoglobulin conjugated to alkaline phosphatase (Promega) for 1 h at 25 °C. The PVDF membrane was subsequently incubated with Western Blue Stabilized Substrate for Alkaline Phosphatase (Promega) for 5 min at 25 °C. Stained image was quantified using ImageJ software.

### Gelatin zymography

Protein samples for zymography were prepared from melanoma cells after the cells were incubated in the RPMI medium (pH 5.5) for 3 h at 25 °C to maintain enzymatic activity. A sample solution containing 10 μg protein was separated by SDS-PAGE.

After SDS-PAGE, the gel was washed with a washing buffer (pH 7.5) containing 0.05 M Tris-HCl, 2.5 v/v% Triton X-100, 5 mM CaCl_2_, 1 μM ZnCl_2_, and 0.016% NaN_3_, for 30 min. Gel was stained with CBB and decolorized with a methanol-acetic acid solution. Band images were quantified using ImageJ software.

### Enzyme-linked immunosorbent assay (ELISA)

TGF-β1 in culture supernatants of BL6 and *Nanog*^+^BL6 cells were measured by ELISA (R&D Systems). Briefly, melanoma cells (6 × 10^4^ cells/well) in 6-well plates were incubated in serum-free R10 medium. After 48 h of incubation, TGF-β1 production in the culture supernatants was measured.

### Count of the number of metastatic colonies and estimation of their volume

PBS (250 μL) containing 2.5 × 10^5^ cells was injected into a mouse tail vein. After two weeks, mice were euthanized by cervical dislocation, and lung and liver tissues were removed, washed with PBS, and incubated in 4 w/v% paraformaldehyde for 18 h at 4 °C for fixation. According to preliminary experiments, no metastasis of BL6 was observed in liver. In this study, however, some colonies might happen to appear because of the effect of *Nanog*. Then, we removed liver as well as lung for analysis. The tissues were immersed sequentially in PBS solutions containing 10, 20, and 30 w/v% sucrose, respectively, for 24 h in this order for dehydration. The weight of each tissue was measured and tissues were cut into several lobes. Each lobe was observed with a microscope, and the volume of every metastatic colony was determined. The shape of each metastatic colony was approximated as a spheroid and the following formula was used: *V* = (1/6) π*ab*^2^, where *a* and *b* were the longest and shortest diameters, respectively.

### Statistics

Specific details regarding statistical analyses are presented in the figure legends. Test sample preparation of mRNA or protein was done using one test sample per dish. Each test sample was analyzed twice, and the average of the two results was recorded as the value for one test sample. In most cases, three test samples were analyzed per analytical item. Results are presented as mean ± standard deviation (SD) or mean ± standard error of mean (SEM). Outliers were determined by a Smirnoff-Grubbs test to be greater than 0.05 two-tailed probability. The statistical significance between two specific data groups was analyzed by paired or unpaired two-tailed Student’s *t* test. The statistical significance of results is denoted by a *p* value or by marking with asterisk(s): ^***^: *p* < 0.001, ^**^: *p* < 0.01, ^*^: *p* < 0.05, ^†^: *p* < 0.1.

## Results

### *Nanog* overexpressing BL6

**Fig. 1:**
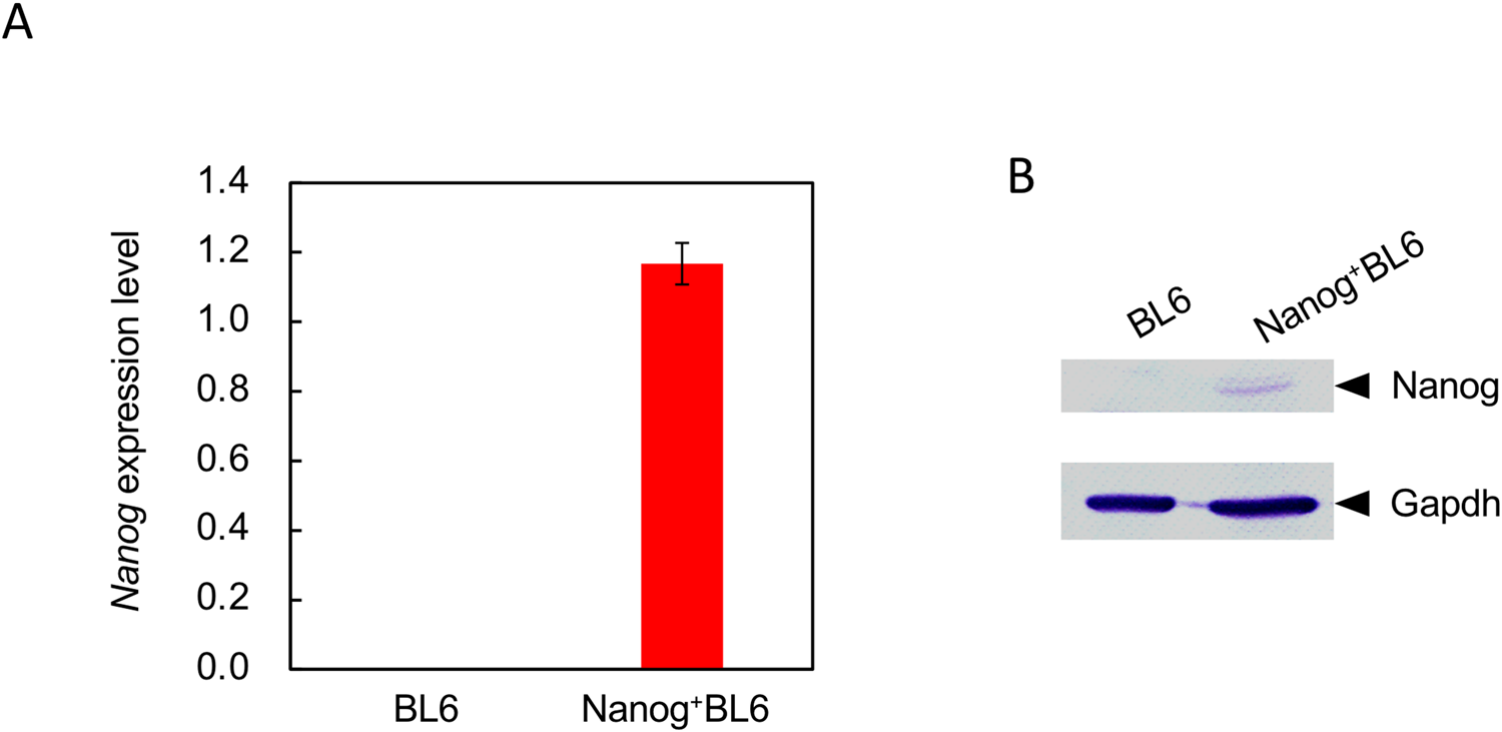
Relative expression of *Nanog* analysis. **A** Expression of mRNA of *Nanog*. Blue coloured rectangle: BL6, red coloured rectangle: *Nanog*^+^BL6. Internal standard: *Gapdh*. Bars are mean ± SD, for *n* = 3. **B** *Nanog* protein expression detected by western blot analysis

*Nanog* is expressed in embryonic stem cells but not in melanoma cells. The overexpression of *Nanog* might influence the growth behavior of BL6 cells. The expression of mRNA and protein of *Nanog* was confirmed by RT-PCR and western analysis, respectively (Fig. 1).

### Increase in the cell proliferation rate

**Fig. 2:**
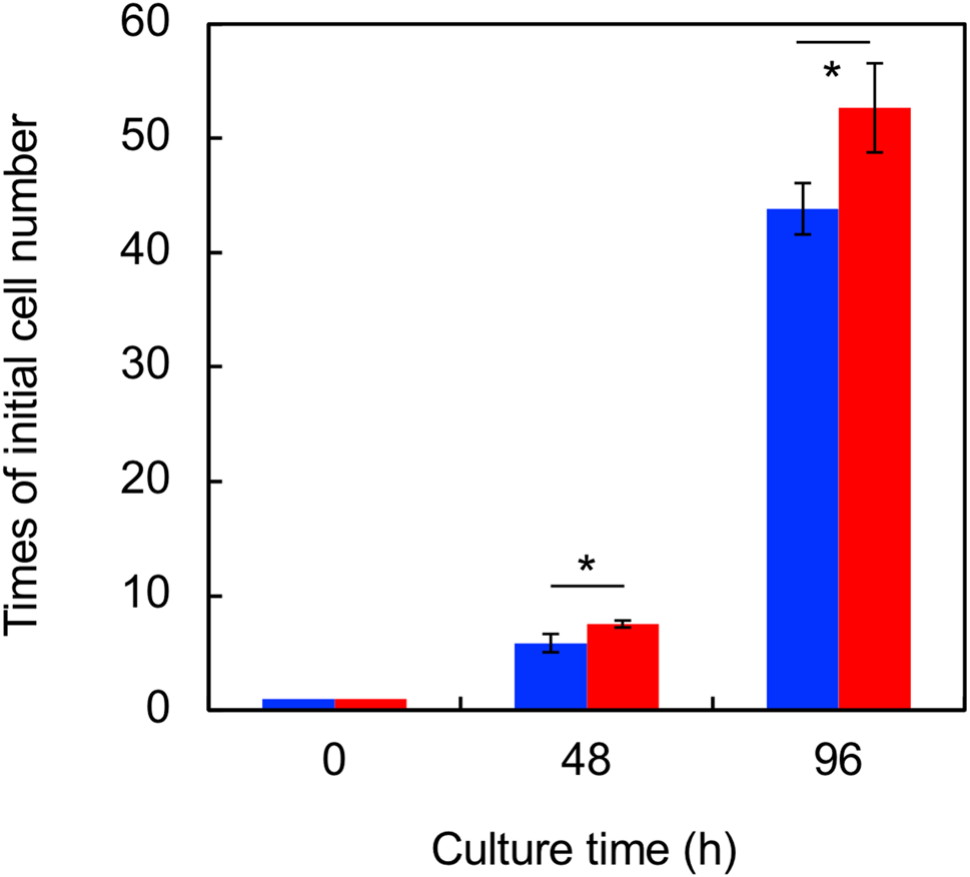
Effect on the cell proliferation. Blue coloured rectangle: BL6, red coloured rectangle: *Nanog*^+^BL6. Bars are mean ± SD, for *n* = 3

The cell proliferation rate is one of the cancer metastasis-related properties of in vitro tests. The number of BL6 cells increased 43.8 × after 96 h (Fig. 2). In contrast, *Nanog*^+^BL6 cells showed a higher growth rate than BL6 cells. The number of *Nanog*^+^BL6 cells was 52.7 × after 96 h.

### Increase in wound-healing activity

**Fig. 3:**
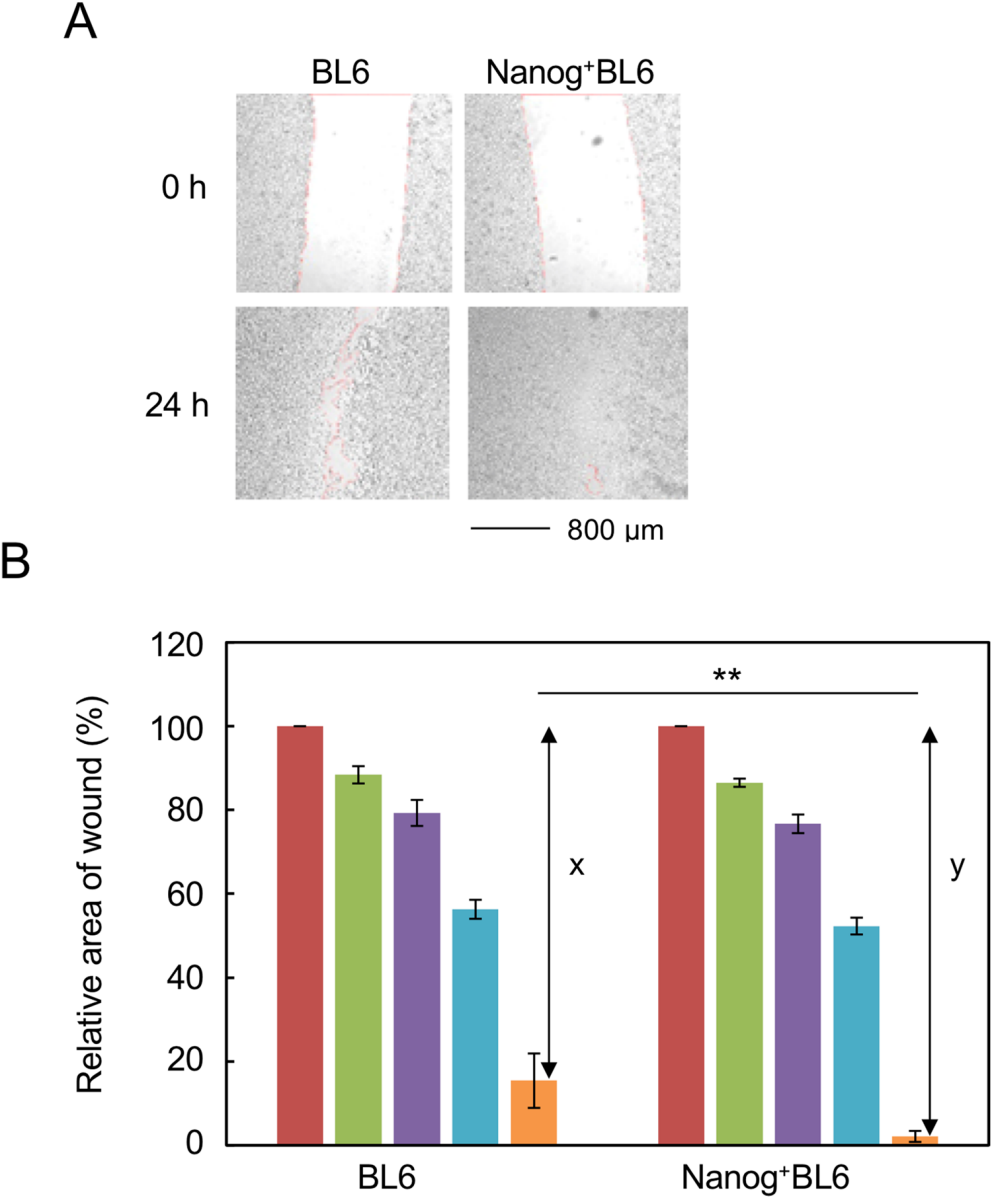
Effect on the wound healing activity. **A** Microscopic images of wound area. **B** Quantification of wound area, brown coloured rectangle: 0 h, green coloured rectangle: 2 h, violet coloured rectangle: 6 h, blue coloured rectangle: 12 h, orange coloured rectangle: 24 h. *x*: 100-BL6 (at 24 h), *y*: 100-*Nanog*^+^BL6 (at 24 h), Enhancement rate: *y*/*x*. Bars are mean ± SD, for *n* = 4

An approximately 800-μm wide scratch was made with a sterile micropipette tip in the cell layer. The healing of the wound by growing melanoma cells was then measured (Fig. 3A). The growing edge of the peeled area formed a complex line. The wound area was measured using ImageJ software and plotted (Fig. 3B). The initial area measured at 0 h was set as 100% in order to determine the wound-healing activity in a 24-h test. We found that the wound healing activity of BL6 was 15.4 (%/24 h). The effect of *Nanog* overexpression was evaluated by the relative value of the wound healing activity of *Nanog*^+^BL6 versus that of BL6. The enhancement rate was 1.16 × , which was given by *y*/*x* illustrated in Fig. 3B.

### Increase in the activities of MMP2 and MMP9

MMPs are a family of 25 zinc-binding matrix proteinases and play an active role in the invasion of cancer cells to target tissues [10]. Particularly, a group of gelatinases such as MMP2 and MMP9 may play a principal role in activating the invasion by specific hydrolysis of type IV collagen in basement membrane [11]. Therefore, this study focuses the involvement of MMP2 and MMP9.

**Fig. 4:**
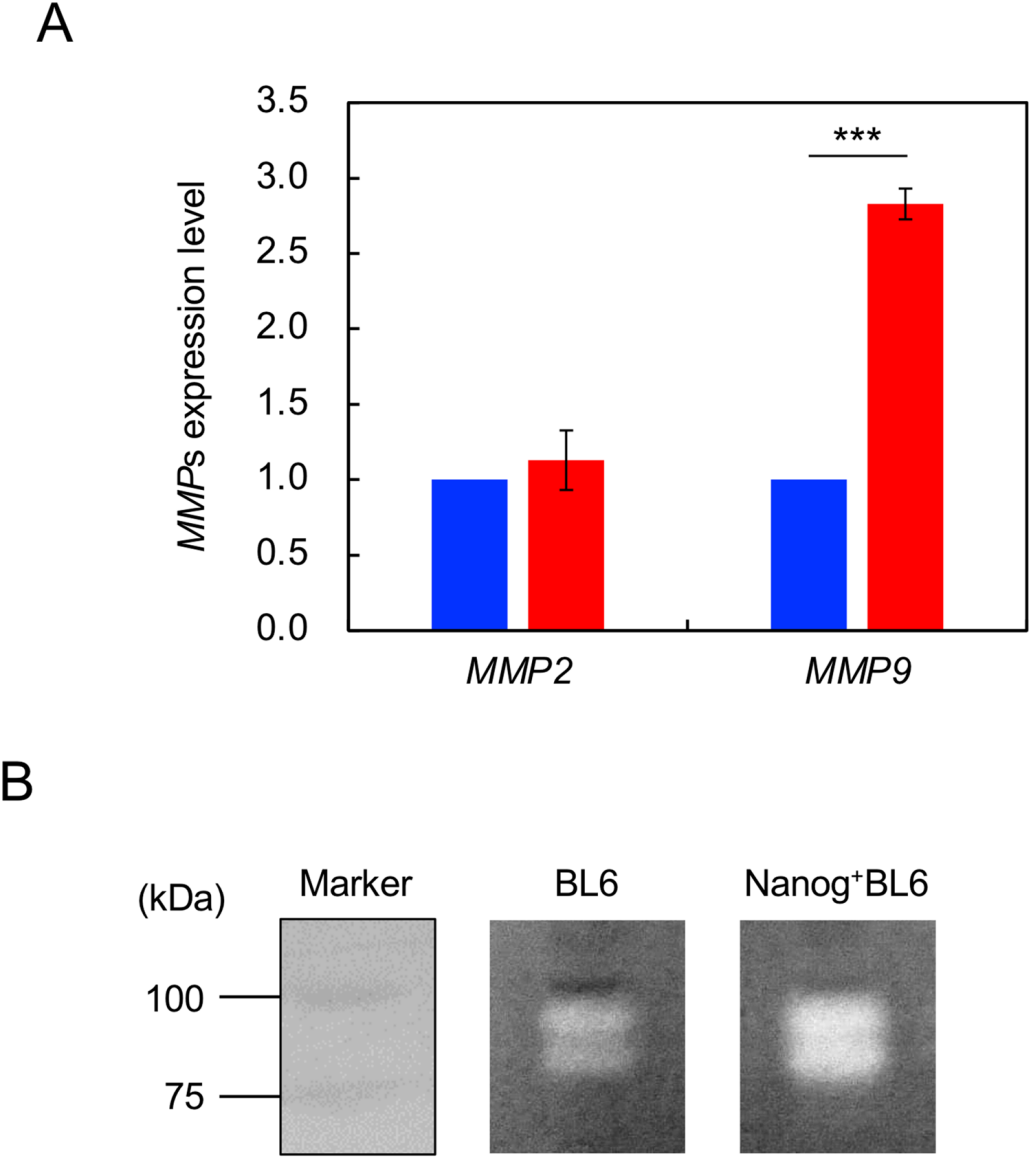
Effects on the expression of MMP2 and MMP9. **A** Expressions of mRNA of *MMP2* and *MMP9*. Blue coloured rectangle: BL6, red coloured rectangle: *Nanog*^+^BL6. Internal standard: *Gapdh*. Bars are mean ± SD for *n* = 3. **B** Zymography of MMP9 in BL6 and *Nanog*^+^BL6

The expressions of *MMP2* and *MMP9* were investigated in *Nanog*^+^BL6 and BL6. The effect of *Nanog* overexpression was observed only on *MMP9* expression, causing a significant increase to 3.2 × greater (*p* < 0.001) (Fig. 4A). The enzyme activity of MMP was confirmed by zymography; quantitatively higher activity was observed in *Nanog*^+^BL6 cells than in BL6 (Fig. 4B). Thus, *Nanog* overexpression increased the enzymatic activity of MMP9.

### Comprehensive expression behavior revealed by transcriptome sequencing

**Fig. 5:**
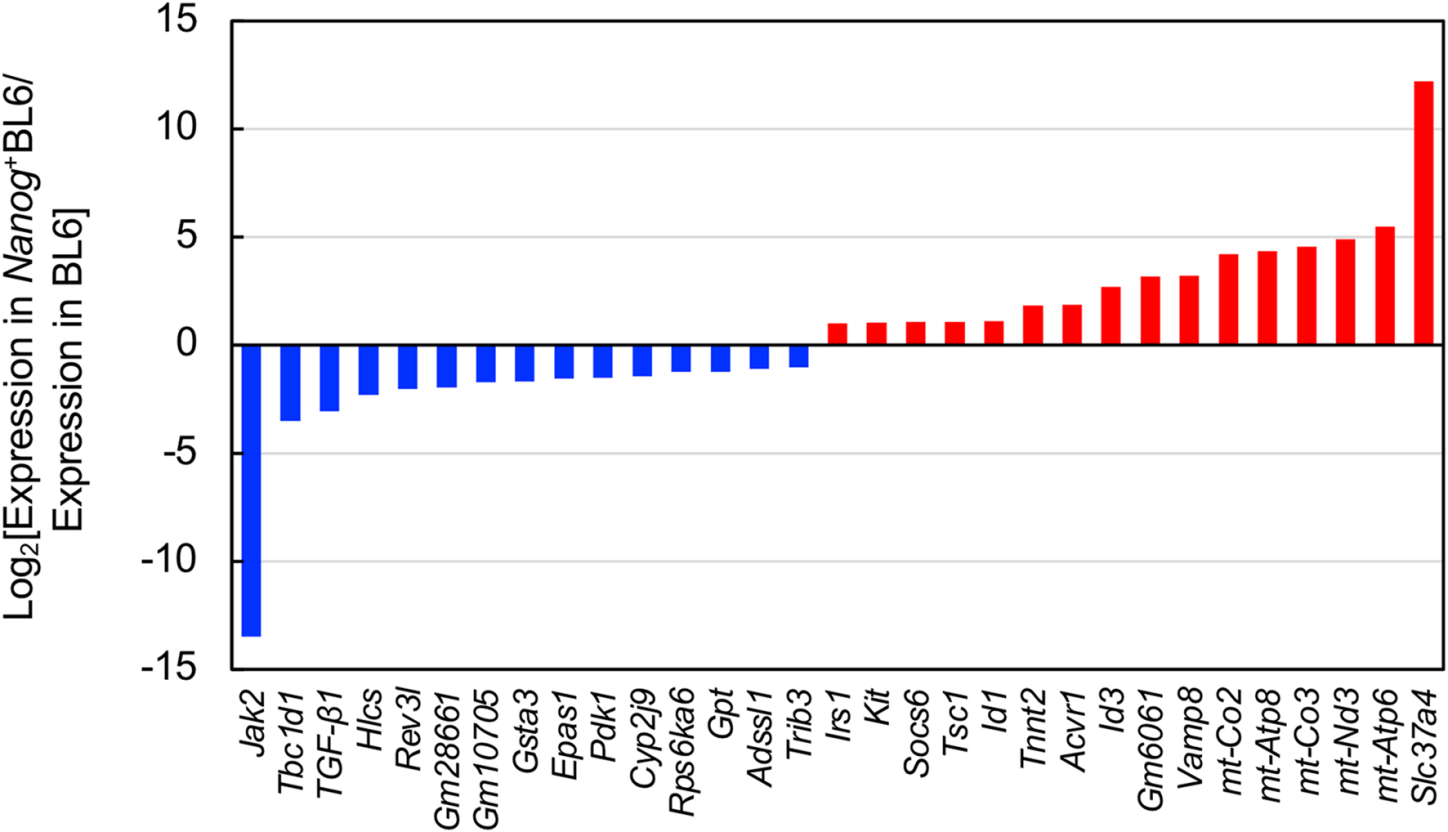
Differential expression of *Nanog*-dependent genes. Significantly changed genes. Red coloured rectangle: up-regulation by higher than 2, blue coloured rectangle: down-regulation by lower than 1/2

Differential gene expression in *Nanog*^+^BL6 versus in BL6 was analyzed by transcriptome sequencing. The number of up-regulated and down-regulated genes was 150 and 172, respectively. Top 15 up-regulated genes and top 16 down-regulated genes were determined (Fig. 5). Three genes (*Akt3*, *Srp54b*, *Large1*) showed up- and down-regulated cases because of multiple RNA fragments with different properties. Potential involvement of these 34 genes in important biochemical metabolic pathways and signal transduction pathways was suggested by KEGG enrichment analysis.

The highest up-regulated gene was *Slc37a4* (solute carrier family 37 member 4; same as glucose-6-phosphatase glucose translocase) that could facilitate vesicle-aided glucose transport from cytosol to endoplasmic reticulum, possibly leading to the activation of glucose metabolism. Second–sixth highest up-regulated genes were *mt-Atp6*, *mt-Nd3*, *mt-Co3*, *mt-Atp8*, and *mt-Co2*. These are speculated to be involved in oxidative phosphorylation. On the other hand, the lowest down-regulated gene was *Jak2*. The suppression of *Jak2* should lead to immunosuppression and apoptosis induction via *Smad2/3*. The second and third lowest down-regulated genes were *Tbc1d1* and *TGF-β1*, respectively. *Tbc1d1* was speculated to be involved in AMPK signaling pathway and transporter-aided glucose uptake. Their suppression should lead to immunosuppression, growth inhibition, and apoptosis of by-standing cells via cytokine secretion. Among these genes, the down-regulation of *TGF-β1* was a marked finding, because *TGF-β1* has been well discussed about its suppressive/progressive dual role in cancer cell growth and metastasis.

### Confirmation of *TGF-β1* down-regulation

**Fig. 6:**
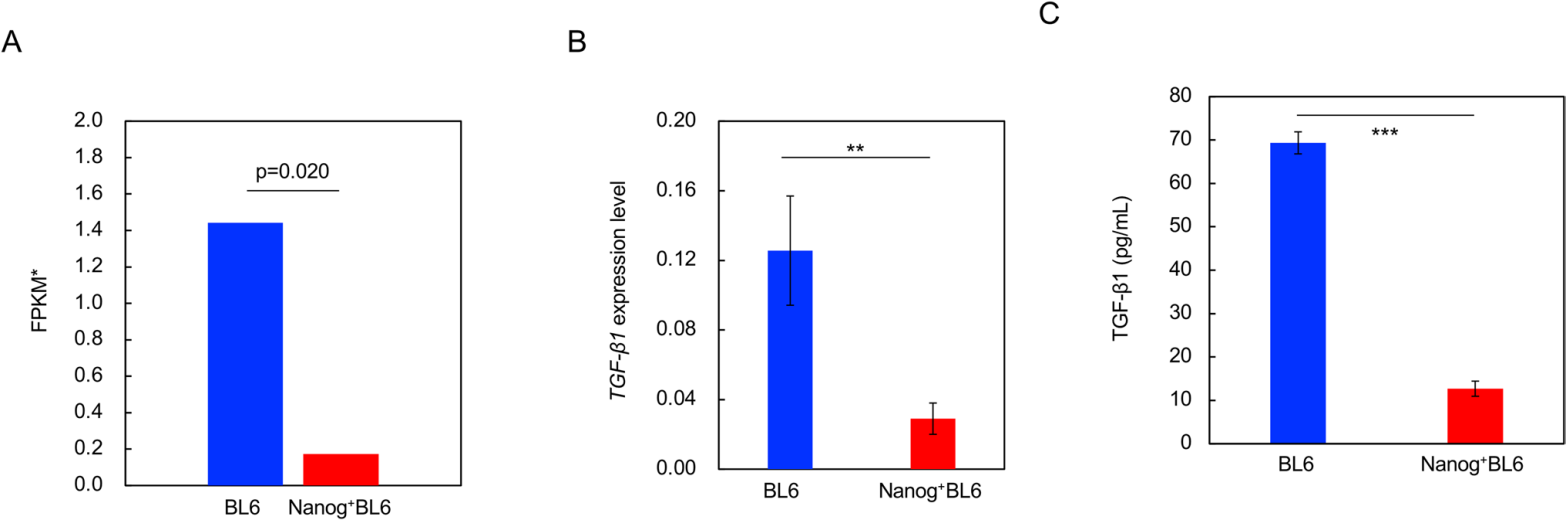
Relative expression of *TGF-β1* analysis. **A** Expression of *TGF-β1* in BL6 and *Nanog*^+^BL6 determined by RNA sequence analysis. Blue coloured rectangle: BL6, red coloured rectangle: *Nanog*^+^BL6. ^*^Fragments per kilobase of exon per million mapped fragments. **B** Expression of *TGF-β1* mRNA in BL6 and *Nanog*^+^BL6 determined by qRT-PCR. Blue coloured rectangle: BL6, red coloured rectangle: *Nanog*^+^BL6. Internal standard: *Gapdh*. Bars are mean ± SD, for *n* = 3. **C** The amount of TGF-β1 secretion in the culture supernatant of cells. Blue coloured rectangle: BL6, red coloured rectangle: *Nanog*^+^BL6. Bars are mean ± SD, for *n* = 3

The result of *TGF-β1* down-regulation based on transcriptome sequencing indicated (Fig. 6A) was confirmed by qRT–PCR (Fig. 6B) and by ELISA. Since TGF-β1 was a secretory cytokine, the culture supernatant of cells was measured by ELISA. The amount of TGF-β1 secretion was greatly reduced in *Nanog*^+^BL6 cells in comparison with BL6 cells (Fig. 6C). Therefore, the down-regulation of *TGF-β1* by *Nanog* overexpression was confirmed.

### Effects of hypoxic condition

**Fig. 7:**
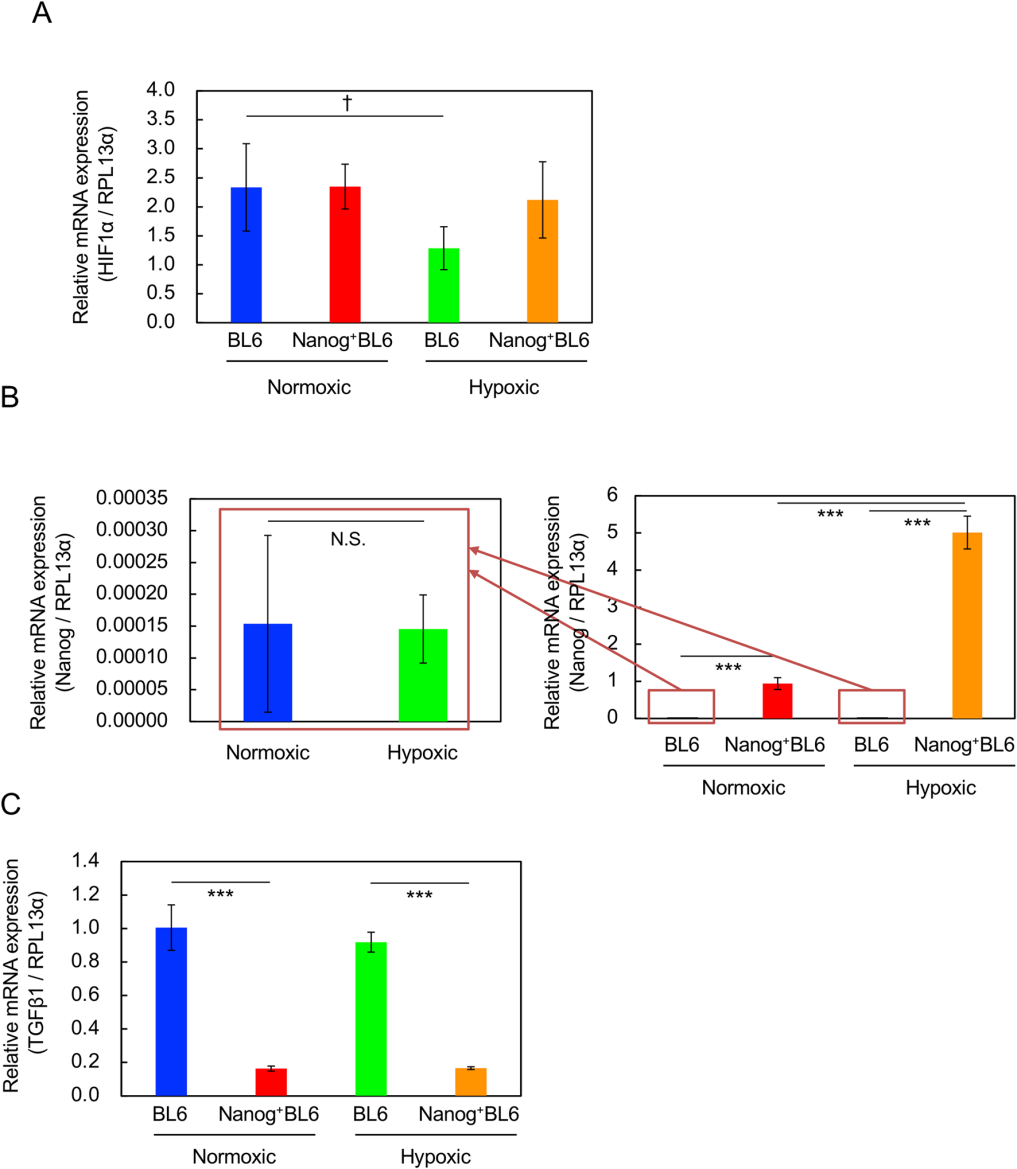
Effects of hypoxic culture condition on the expression of relevant genes determined by qRT-PCR. Internal standard: *RPL13α* according to [22]. Blue coloured rectangle: BL6 under normoxic condition, red coloured rectangle: *Nanog*^+^BL6 under normoxic condition, green coloured rectangle: BL6 under hypoxic condition, orange coloured rectangle: *Nanog*^+^BL6 under hypoxic condition. **A** *HIF-1α*. **B** *Nanog*. N.S.: no statistical significance. **C** *TGF-β1*. Bars are mean ± SD, for *n* = 3

The gene expressions of *HIF-1α* (hypoxia inducible factor), *Nanog*, and *TGF-β1* were investigated under hypoxic culture condition. The gene expression of *HIF-1α* was down-regulated in wild BL6 cells (Fig. 7A). Under hypoxic condition, however, *HIF-1α* protein should increase and then its gene expression might be suppressed by negative feedback control [12]. In *Nanog*^+^BL6 cells, however, no change in *HIF-1α* was observed. *Nanog* gene expression was insensitive to hypoxic condition in BL6 cells, though its level was very low (Fig. 7B). In *Nanog*^+^BL6 cells, however, the overexpression level of *Nanog* under hypoxic condition was higher than under normoxic condition.

*TGF-β1* gene expression was down-regulated by *Nanog* overexpression but insensitive to hypoxic condition in both BL6 and *Nanog*^+^BL6 cell lines (Fig. 7C). These results indicate that the down-regulation of *TGF-β1* should be caused by *Nanog* overexpression as long as the *Nanog* expression level is high enough. That might not be due to unusual culture conditions such as hypoxic condition.

### Metastasis of *Nanog* overexpressing cells

**Fig. 8:**
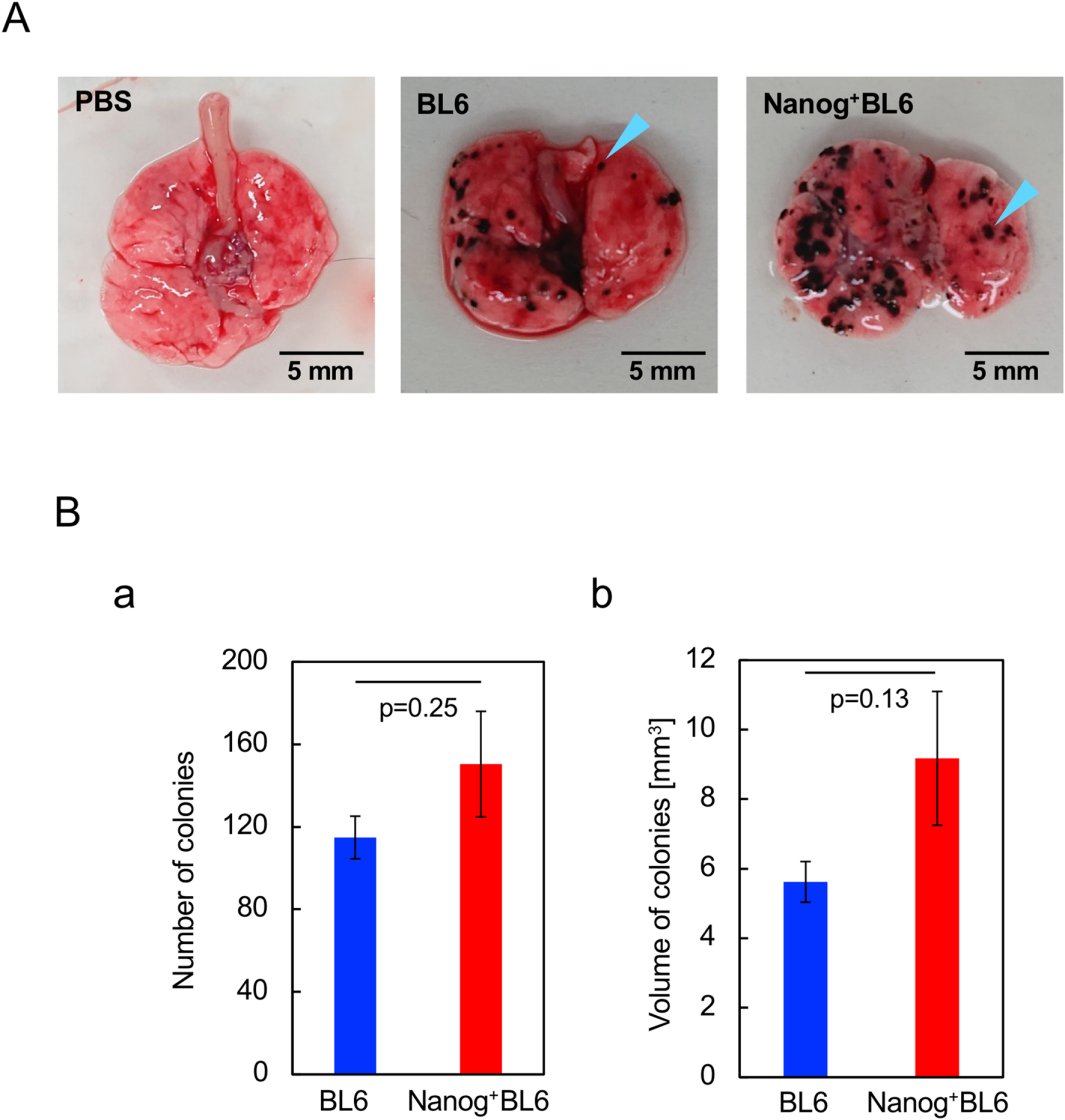
Metastatic colonies generated in lung. **A** Photographs of dissected lung tissue. A blue arrowhead indicates an example of a single colony. **B** Total number and volume of metastatic colonies. Number of mice tested: six for BL6 and seven for *Nanog*^+^ BL6. Statistical significance was indicated by *p *value

Lungs and other organs were removed two weeks after mouse tail injection of test cells. Test cells were BL6 and *Nanog*^+^BL6. Black colonies of different sizes were detected in most samples of lungs (Fig. 8A) but not in any other organs. Colony size was distributed from 0.1 to 1.0 mm in diameter. Since colony shape was not necessarily a sphere, colony volume was determined by the formula for a spheroid. The smallest colony had a size of approximately 90 μm and a volume of 0.0004 mm^3^. Though statistical significance was poor, there appeared a tendency that both number and volume of colonies in *Nanog*^+^BL6 were 1.31 × and 1.63 × greater than those in BL6 (Fig. 8B). Therefore, the effect of *Nanog* overexpression was progressive rather than suppressive even on the metastasis of BL6.

## Discussion

Previously we investigated the role of undifferentiated state-related genes in mouse embryonic stem cells. The undifferentiated state is understood to be maintained by dynamic expression balance of *Oct3/4*, *Nanog*, *Sox2*, and other factors [13, 14]. Under such a balance, leukemia inhibitory factor (LIF) is also essential for the complete maintenance of undifferentiated state [15]. When they lose balance, differentiation is thought to be initiated. To our surprise, however, the microinjection of an *Oct3/4*- or *Nanog* overexpressing vector into ES cells could maintain undifferentiated state for 72 h under an unbalanced condition even in the absence of LIF [16, 17]. Therefore, the overexpression of *Nanog* alone could be effective to change the cell state of melanoma cells to a more undifferentiated state and then effective to enhance the metastatic potential. Consequently, our expectation was supported by in vitro and in vivo tests using a melanoma cell line BL6.

The more the undifferentiated state of cancer cells, the higher the metastatic potential. Such a suggestion should have come from the concept of cancer stem cells (CSCs) [18, 19]. This led to the search for CSC markers in various cancers such as melanoma [20], colorectal [21], and breast cancers [22] in the development of therapies targeting CSCs. The specificity and effectiveness of the marker candidates so far found, however, are still insufficient for the quantitatively strict definition of CSC. Similarly, it might be still unclear whether *Nanog*^+^BL6 is a CSC, because the expression of CSC markers such as HCAM and ABCB5 could not be detected.

Signaling pathways following *Nanog* expression vary depending on the types of cancer cells. The enhancement of *Nanog* expression promoted epithelial–mesenchymal transition (EMT) through activation of the *Stat3*/*Snail* signaling system in liver cancer cells [23]. The promotion of EMT is well understood to facilitate tumor growth and the involvement of MMPs in EMT has been well remarked. In ovarian cancer, two pathways of sugar metabolism are involved in cancer cell growth and mobility: *HK2* expression → *FAK* → *ERK1/2* → *Nanog* → stem cell growth enhancement, and *ERK1/2* → *MMP9* → increased cell mobility [4], where FAK refers to focal adhesion kinase and ERK refers to extracellular signal-related kinase. It is curious that factors referred to in former reports were almost not demonstrated as *Nanog*-induced genes by KEGG enrichment analysis except for *TGF-β1*.

TGF-β1 is well known as a cytokine and frequently observed as a multi-functional factor, promoting tumor growth under some conditions, while suppressing it under other conditions. An interesting study was reported formerly. In B16-F10 melanoma cells under hypoxia, *Nanog* expression was up-regulated, *TGF-β1* expression was then enhanced and finally immunosuppression and increased regulatory T cells were induced in tumors and macrophages [7]. This report suggested that the up-regulation of *TGF-β1* could enhance the growth of cancer cells and metastasis as well. In contrast, however, our results demonstrated that the increase in *Nanog* expression caused the down-regulation of *TGF-β1* but eventually promoted metastatic colony growth. The role of TGF-β1 in signaling from *Nanog* to tumor growth regulation seemed to be opposite. We suspect that one of the reasons for this inconsistency may be the difference in *Nanog* expression levels. In fact, the *Nanog* expression level in wild BL6 under hypoxic condition was much lower than that in *Nanog*^+^BL6 (Fig. 7B). Our study has clarified, for the first time, that *Nanog* overexpression-induced down-regulation of *TGF-β1 *can enhance the metastatic potential of melanoma cell lines. This finding, however, may not be surprising, given the multi-functionality of *TGF-β1*.

Another point to be discussed was the involvement of MMP. In the case of A375 human melanoma cells, MMP was down-regulated by *Nanog* overexpression [1], though the MMP was a membrane type 1 (MT1-MMP) and different from a secretion type MMP9. Here, our result has added a novel case of MMP9 up-regulation in mouse melanoma cells to *Nanog* signaling.

The effectiveness of *Nanog* overexpression in enhancing the metastatic potential of melanoma BL6 cell lines has been demonstrated for the first time by in vitro and in vivo tests. The down-regulation of *TGF-β1* was involved in increased metastasis by *Nanog* overexpression. The results of this study will once again shed light on *TGF-β1* as a clinical target.

## Supplementary Information

Below is the link to the electronic supplementary material.

Supplementary material 1 (XLSX 11 kb)
